# Association between Milk Consumption and Metabolic Syndrome among Korean Adults: Results from the Health Examinees Study

**DOI:** 10.3390/nu9101102

**Published:** 2017-10-08

**Authors:** Sangah Shin, Hwi-Won Lee, Claire E. Kim, Jiyeon Lim, Jong-koo Lee, Daehee Kang

**Affiliations:** 1 Department of Food and Nutrition, Chung-Ang University, Gyeonggi-do 17546, Korea; ivory8320@cau.ac.kr; 2 Department of Preventive Medicine, Seoul National University College of Medicine, Seoul 03080, Korea; hwiwon@snu.ac.kr (H.-W.L.); claireekim@snu.ac.kr (C.E.K.); jiyeonlim@snu.ac.kr (J.L.); 3 Department of Biomedical Sciences, Seoul National University Graduate School, Seoul 03080, Korea; 4 JW Lee Center for Global Medicine, Seoul National University College of Medicine, Seoul 03087, Korea; docmohw@snu.ac.kr; 5 Department of Family Medicine, Seoul National University Hospital, Seoul 03080, Korea; 6 Institute of Environmental Medicine, Seoul National University Medical Research Center, Seoul 03080, Korea

**Keywords:** metabolic syndrome, milk, the Health Examinees (HEXA) study, Korean

## Abstract

It has been suggested that a greater dairy consumption, particularly of milk, may have contributed in lowering the prevalence of metabolic syndrome (MetS). A cross-sectional analysis was conducted to examine the association between milk consumption and MetS, and its components among Korean adults aged 40–69. A total of 130,420 subjects (43,682 men and 86,738 women) from the Health Examinees Study were selected for the final analysis. Milk consumption was estimated using a validated 106-item food frequency questionnaire. MetS was defined using the National Cholesterol Education Program, Adult Treatment Panel III (NCEP III). Logistic regression analyses were performed to calculate the odds ratios (ORs) and 95% confidence intervals (CIs) between milk consumption and MetS after adjusting for potential confounders. In this study, the average milk consumption was 77.9 g/day, with the overall prevalence of MetS being 26.1% (29.1% in men and 24.6% in women). We found that the prevalence of the MetS was significantly lower in subjects with higher milk consumption (*p* < 0.0001). Adjusted OR for MetS was significantly lower in the highest milk consumption category (≥1 serving/day among men; ≥2 serving/day among women) than those in the lowest milk consumption category (OR: 0.92 95%CI: 0.86–0.99, *p* trend = 0.0160 in men; OR: 0.68, 95%CI: 0.60–0.76, *p* trend < 0.0001 in women). Overall, higher milk consumption was inversely associated with the MetS components: elevated waist circumference, elevated triglyceride, and reduced high-density lipoprotein cholesterol (HDL-C) (all *p* trend < 0.05). This study concludes that higher milk consumption is associated with the lower odds of MetS in Korean adults.

## 1. Introduction

Metabolic syndrome (MetS), characterized by a cluster of metabolic disturbance, including abdominal obesity, insulin resistance, hyperglycemia, dyslipidemia, and hypertension, is a well-known risk factor for type 2 diabetes mellitus and atherosclerotic cardiovascular disease [1]. Additionally, a recent meta-analysis reported that MetS is associated with an increased risk of common cancers, including colorectal and breast cancer [2]. With an increase in obesity and sedentary lifestyle globally, the prevalence of MetS has increased around the world [3]. Moreover, the prevalence of MetS in Korea has also increased, especially during the last twenty years or so, 19.6% in 1998 to 28.9% in 2013 [4]. Therefore, now more than ever, MetS is an important target for public health intervention. Although the etiology of MetS is largely unknown, it is thought that genetic, metabolic, and environmental factors, such as diet and physical activity, all play an important role in its development [5]. In recent years, numerous studies have reported the benefits of milk or dairy consumption with regard to lowered risk of (individual) MetS components, such as dyslipidemia [6], hyperglycemia [7], type 2 diabetes [8], hypertension [9], and abdominal obesity [10].

Numerous cross-sectional and prospective studies from the United States (US) and Europe have reported that dairy product consumption may contribute to a decreased risk of developing MetS by affecting one or more of its components, such as weight gain, blood pressure, lipid levels, and insulin sensitivity [11,12,13,14]. In addition to the studies conducted in Western countries, similar studies have been conducted in Asian countries. A study conducted among 59,796 middle-aged and older Japanese reported an inverse association between dairy product consumption and type 2 diabetes mellitus only in women [15]. Prospective studies from China have suggested that dairy consumption was associated with a significantly lower risk of type 2 diabetes and favorable changes of cardiometabolic traits in China [16]. A recent meta-analysis of nine prospective studies and 12 cross-sectional studies performed in Western and Asian countries indicated that dairy consumption is inversely associated with both incidence and prevalence of MetS [17]. Although a beneficial effect of dairy consumption has been repeatedly reported, other observational studies are noted otherwise [18,19]. 

As dairy products are generally not included in the traditional Korean diet, the consumption of milk, yogurt, and cheese is relatively lower than that of the Western countries [20]. Only a handful of evidence suggests that increased dairy product intake may be protective against MetS among the Korean population [21,22]. Most of the previous studies on dairy consumption with MetS were conducted among Western countries. Moreover, most studies from Korea were conducted with a small size population (<10,000 subjects). Therefore, there are still limited evidence of the association between dairy products and MetS among the Korean population. Considering that the prevalence of MetS is increasing dramatically in Korean adults [23], understanding the association between the risk of MetS and milk consumption is crucial to prevent MetS. Therefore, we attempted to examine whether higher dairy products may reduce the prevalence of MetS and its components using large-scaled Korean population data (>130,000 subjects). Accordingly, the present study aimed to investigate the impact of milk consumption on the MetS and its components among Korean adults aged 40–69 in the Health Examinees (HEXA) study.

## 2. Subjects and Methods

### 2.1. Study Population

This study was based on a large-scale genomic cohort study, the Health Examinees (HEXA) Study. For a baseline, a total of 169,722 subjects aged between 40–69 years old were recruited via 38 general hospitals and health examination centers throughout Korea between 2004 and 2013. The HEXA study design has been described in detail in previous studies [24,25]. 

**Figure 1:**
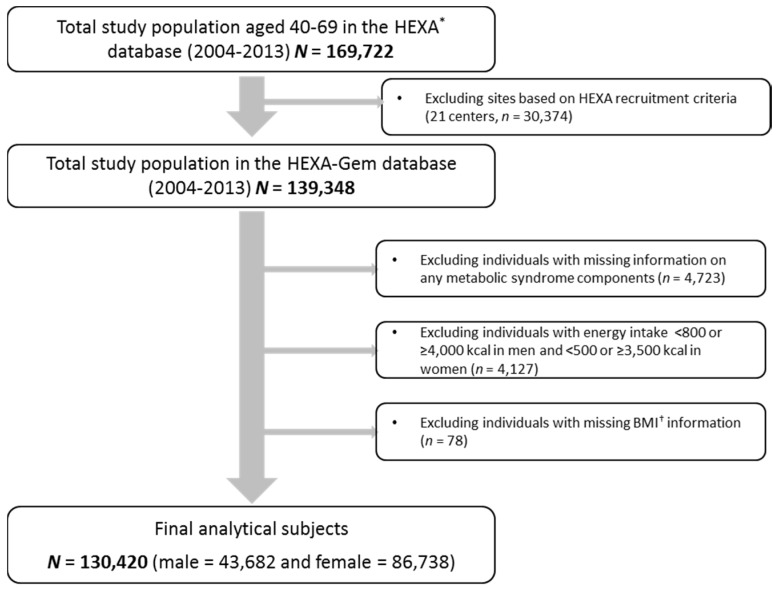
Flow diagram of analytical sample. * HEXA: Health Examinees, ^†^ BMI: Body Mass Index.

Updated from the previously published HEXA studies, the current study uses the HEXA-G (Health Examinees-Gem) participant sample which was given additional eligibility criteria on the participating sites (i.e., health examination centers and training hospitals). Of the original 38 sites, HEXA-G applied exclusion criteria of the following: 8 sites (*n* = 9370) that only participated in the pilot study years 2004–2006; 8 sites (*n* = 12,205) that did not meet the HEXA biospecimen quality control criteria (i.e., different testing protocols); and 5 sites (*n* = 8799) that have participated in the study for less than two years. In the new HEXA-G data, a total of 139,348 participants remained. Among in HEXA-G subjects, a total of 7274 were excluded: missing information on any MetS components at baseline survey (*n* = 4723); energy intake <800 or ≥4000 kcal/day in men and <500 or ≥3500 kcal/day in women (*n* = 4127); and missing information on Body Mass Index (BMI) (*n* = 78). Ultimately, 130,420 subjects, including 43,682 men and 86,738 women, remained in the final analysis (Figure 1). All of the participants voluntarily signed an informed consent form. This study was approved by the Ethics Committee of the Korean Health and Genomic Study of the Korean National Institute of Health, and the institutional review boards of all participating hospitals (IRB No. E-1503-103-657).

### 2.2. Assessment of Milk and Dairy Food Consumption

The self-administered food frequency questionnaire (FFQ) assessed the consumption of 106 food and beverage items during the past year. Dairy products include milk, yogurt, and cheese. Food consumption frequency categories were as follows: never or almost never, once per month, two to three times per month, one to two times per week, three to four times per week, five to six times per week, once per day, two times per day, and three times per day. Portion sizes were also measured: one-half standard serving, one standard serving, and one and a half. One serving is equal to 200 mL of milk, 130 mL drinking yogurt, and 20 g of cheese.

The amount of milk consumption was converted to weekly frequencies and then multiplied by the reported portion sizes for each food. To categorize milk consumption, we considered the data distribution of milk consumption. Among milk consumers in our study, the average consumption of milk in women was higher than that of men (115.8 g/day in women vs. 96.1 g/day in men). The number of subjects in category of ≥2 servings/day among women was 2606 (3.0%), which was an adequate number of subjects for statistical analysis; however, among men, there were only 851 subject (1.9%). Consequently, we categorized women into five groups (none or rarely, <3 servings/week, 3 ≤ to < 7 servings/week, 1 serving/day, and ≥2 servings/day); men into four groups (none or rarely, ≤2 servings/week, 3–6 servings/week, and ≥1 serving/day) according to milk consumption. Total energy and nutrient intake was calculated via a food composition table that was developed by the Korean Health and Industry of Development Institute [26]. The test for validity and reproducibility of our FFQ has been performed in a previous study [27]. Regarding validity, age, sex, and energy intake, adjusted correlation coefficients between the FFQ and the 12-day DRs ranged between 0.23 and 0.64 (median for all nutrients 0.39). Regarding reproducibility, the averages of correlations between the two FFQs one year apart were 0.45 for all nutrient intake and 0.39 for nutrient densities [27].

### 2.3. Definition of Metabolic Syndrome

The current study defined metabolic syndrome using the National Cholesterol Education Program Adult Treatment Panel III (NCEP-ATP III) [28], modified according to Asian guideline for waist circumference. Participants who met three or more of the following criteria were classified as having metabolic syndrome: (1) waist circumference (WC) ≥90 and ≥80 cm for men and women, respectively; (2) triglycerides (TG) ≥150 mg/dL or drug treatment for elevated triglycerides; (3) high-density lipoprotein cholesterol (HDL-C) ≤40 and ≤50 mg/dL in men and women, respectively; (4) systolic blood pressure (BP) ≥130, diastolic blood pressure ≥85 mmHg or drug treatment for elevated blood pressure; and, (5) fasting glucose ≥100 mg/dL or drug treatment for elevated fasting blood glucose.

### 2.4. Covariates

Sociodemographic factors such as age, education, and health-related behavior were included. Age was categorized into three groups: 40–49, 50–59, and 60–69 years old. Education was classified into four categories: elementary school or below, middle school, high school graduate, and college or above. Smoking status was defined using the same categories used by a previous study of our research group [29]. Smoking status was ascertained by posing the following question: “Have you smoked more than 20 packs of cigarettes (400 cigarettes) in your lifetime?” People who had smoked ≥400 cigarettes during their lifetime and still smoked cigarettes at the time of the survey were classified as current smoker; Subjects who responded as never having smoked 400 cigarettes were defined as non-smokers, and past-smoker were defined as those who had smoked ≥400 cigarettes during their lifetime but did not smoke at the time of the survey. Alcohol drinking status was determined by the following question: “Are you unable to consume alcohol or refuse to do so (for religious reasons, etc.)?” Respondents who have never drank alcohol were determined as never drinkers. On the other hand, drinkers who have answered the question “Do you still drink” with “yes” were determined as current drinkers and “no” as former drinkers. Physical activity was assessed by posing the following question: “Do you regularly participate (enough to sweat) in any sports?” Subjects who responded “yes” to the question were assigned to the regular exercise group; the respondents who answered “no” were assigned to the non-regular exercise group.

### 2.5. Statistical Analysis

All of the analyses were performed separately by sex to investigate the association between milk consumption and MetS. The chi-square test (for categorical variables) and linear regression (for continuous variables) were used to analyze the characteristics of the study population based on milk intake. A multivariable logistic regression model was used to assess whether milk consumption was independently associated with the odds for prevalent MetS. Multivariable-adjusted model including age, BMI, education, smoking, alcohol drinking, and physical activity as covariates was used. We considered it necessary to adjust for BMI as a mediator due to it being a strong risk factor for MetS and its components. However, as BMI is highly correlated with waist circumference, the final model excluded BMI from its WC OR calculation. The final multivariable model was adjusted for age (40–49, 50–59, and 60–69 years), BMI (continuous; not adjusted for WC), education (≤elementary, middle, high, ≥college, and unknown), smoking (never, past, current, and unknown), alcohol drinking (non, current, and unknown), regular exercisers (yes, no, and unknown), and total energy intake (quartiles). Initially, we have adjusted for the nondairy food groups (i.e., fruit, vegetables, and meat). However, when we performed another analysis excluding the nondairy food groups in the model, the results did not change substantially. Therefore, in pursuing a parsimonious model, we excluded the nondairy food groups in our final model. Moreover, we performed a sensitivity analysis by excluding subjects with a history of type 2 diabetes, hypertension, and dyslipidemia. 

The 95% confidence intervals (95% CIs) of the odds ratios (ORs) were estimated using the Wald method. The test of the linear trend across increasing categories of milk consumption was conducted by assigning the median consumption within each category and as a continuous variable. All of the *p*-values were two-sided, and statistical significance was set at below 0.05. All of the analyses were performed with SAS (version 9.4; SAS Institute Inc., Cary, NC, USA).

## 3. Results

The mean age of subject in current study was 53.6 years in men and 52.3 years in women. The mean dairy food group intakes were 77.4 g/day for milk, 33.5g/day for yogurt, and 0.9 g/day for cheese. The characteristics of the 130,420 subjects from the HEXA study based on milk consumption are presented in Table 1. Among our subjects, 16.5% of men and 23.3% of women consumed ≥1 serving/day of milk while 43.4% of men and 34.6% of women were none or rarely (≤1 serving/month) consumed. Men reporting higher milk consumption (≥1 serving/day) and that had higher education were less likely to be current smokers, and more likely to be regular exercisers when compared with those who consumed none or rarely. Women who consumed ≥2 servings/day of milk were younger, had lower BMI, had higher education, were more likely to be current drinkers, and were regular exercisers as compared to those who consumed none or rarely of milk. Both men and women with higher milk consumption (≥1 serving/day in men and ≥2 serving/day in women) had higher intakes of energy, carbohydrates, protein, fat, calcium, and higher intake of food group (i.e., grain, vegetables, fruit, and meat) than those in the lowest category of milk consumption (none or rarely), whereas energy from carbohydrates was inversely associated with milk consumption.

The prevalence of MetS was 26.1% (29.1% in men and 24.6% in women) among our subjects. The multivariable-adjusted ORs (95% CIs) of MetS and its components across categories of milk consumption are shown in Table 2. After adjusting for potential confounders such as age, BMI, education, smoking, drinking, regular exercise, and total energy intake, men with higher milk consumption (≥1 serving/day) had decreased odds of MetS (OR: 0.92, 95% CI: 0.86–0.99, *p* for trend = 0.0160) when compared with those in the lowest category of milk consumption (none or rarely). Women who consumed ≥2 serving/day of milk had 32% lower odds of MetS (OR: 0.68, 95% CI: 0.60–0.76, *p* for trend <0.0001) as compared to those who consumed none or rarely milk. Higher milk consumption (≥ 1 serving/day in men and ≥2 serving/day in women) was associated with a reduced odds of individual MetS components: elevated WC (OR: 0.91, 95% CI: 0.84–0.99 in men; OR: 0.79, 95% CI: 0.71–0.89 in women), hypertriglyceridemia (OR: 0.91, 95% CI: 0.84–0.99 in men; OR: 0.76, 95% CI: 0.69–0.85 in women), and lower HDL-C (OR: 0.84, 95% CI: 0.79–0.89 in men; OR: 0.61, 95% CI: 0.56–0.67 in women).

Adjusting for dietary factors such as intake of fruit, vegetables, and meat, did not alter our results. (OR: 0.92, 95% CI: 0.86–0.99, *p* for trend = 0.0167 in men and OR: 0.67, 95% CI: 0.59–0.75, *p* for trend < 0.0001 in women). Moreover, excluding subjects with a history of type 2 diabetes, hypertension and dyslipidemia did not affect the association of egg consumption with the odds for MetS (OR: 0.90, 95% CI: 0.82–0.99, *p* for trend = 0.0408 in men and OR: 0.80, 95% CI: 0.75–0.85, *p* for trend < 0.0001 in women).

## 4. Discussion

In this study of healthy Korean men and women aged 40–69, we identified an inverse, linear relationship between milk consumption and the odds of MetS. Regarding MetS components, milk consumption was associated with decrease odds in elevated waist circumference, hypertriglyceridemia, and reduced HDL-C.

To an extent, our results support prior study findings conducted in Korea [20,22], as well as in other countries [30,31] regarding the direction of association between dairy product intake and MetS. Among Korean women aged ≥19 years, subjects who consumed ≥1 serving/day of dairy products had lower risks of MetS than women who did not consume dairy products [21]. In a recent meta-analysis including nine prospective cohort studies, the relative risk of MetS among those with high milk or dairy consumption was 0.85 (95% CI: 0.73–0.98) as compared to those with low consumption [17]. By contrast, some studies have suggested positive or null associations between the intake of dairy products and MetS [32,33]. In the elderly Dutch population, higher dairy consumption was not associated with lower weight or more favorable levels of components of the MetS [33]. In another cross-sectional study including Brazilian adults aged >35 years, no associations were observed between frequent dairy consumption and the prevalence of the MetS [34]. The discordant results from these studies may be due to the differences in the MetS definition, study population, and study design. 

Several potential mechanisms for the association of milk consumption with MetS have been suggested. Various nutrients from milk, including calcium and dairy protein, may synergistically protect against Mets and individual components. Calcium in milk may increase the binding of fatty acids and bile acids in the intestine, thereby increasing fecal fat excretion and/or inhibiting fat reabsorption [35]. Hence, calcium can improve the ratio of HDL-C: low-density lipoprotein cholesterol (LDL-C) [36]. In a previous intervention study, increased calcium intake from milk attenuated postprandial triglyceride level, potentially through reduced fat absorption [37]. Calcium also plays a critical role in the body weight regulation by affecting adipocyte intracellular calcium concentrations and decreasing fatty acid synthesis, but increasing lipolysis, and thus depleting triglyceride stores [12]. Furthermore, dietary calcium reduces blood pressure via the suppression of 1,25-dihydroxyvitamin D, thereby normalizing intracellular calcium in vascular smooth muscle [38].

Other nutrients of milk, such as protein and fatty acids, were identified as protective factors of MetS in observational studies. Casein and whey protein, major proteins in milk, may improve the lipid metabolism by reducing the postprandial triglyceride response [39]. Total milk protein is comprised primarily of casein and whey protein, which constitute ~80 and 20% of the total protein fraction, respectively [40]. Milk protein, particularly whey protein, has been associated with improved serum lipid profile [41] and been shown to positively influence body composition. Whey proteins contain angiotensin converting enzyme (ACE)-inhibiting peptides [42]. Hydrolyzed whey proteins can inhibit ACE in vitro, thereby inducing subsequent inhibition of angiotensin II hormone [42], which upregulates fatty acid synthase expression, leading to adipocyte lipogenesis [43]. Therefore, the inhibition of ACE by whey proteins may lead to decreased endogenous fat production [44]. Even though there were no statistically significant association between milk consumption, blood pressure, and glucose level, previous studies reported beneficial effect of milk consumption on blood pressure [6,22] and glucose level [22]. It may be that milk proteins (i.e., casein and whey protein) regulate blood pressure by inhibiting of angiotensin I-converting enzyme, a potent vasoconstrictor [45]. In a previous intervention study, supplementation with whey and casein proteins resulted in lower blood pressure when compared to that of the control group at 12 weeks [46]. Additionally, fasting triglyceride levels and insulin resistance were significantly lowered in the whey protein group as compared with the control group at 12 weeks [41]. 

The milk fat is considered a saturated fat, but it contains also a large amount of oleic acids as well as short-chain and medium-chain fatty acids [47]. Medium-chain fatty acids reduce fat mass in rodents via the down-regulation of adipogenic genes and reduced lipoprotein lipase activity [48]. Approximately 25% of milk fat is made up of the mono-unsaturated fatty acid (MUFA) oleic acid, making oleic acid the second most abundant fatty acid in milk fat [40]. MUFA-rich diets have beneficial effects on the ratio of HDL-C: total cholesterol; and, also tend to reduce triglyceride less but elevate HDL-C [49]. These may be due in part to reduced stimulation of cholesteryl ester transfer protein, a key enzyme in reverse cholesterol transport [50]. 

Among our subjects, it should be noted that the inverse association between milk intake and MetS may be contributed to the overall healthier dietary pattern associated with higher milk intake. In our finding, people with a higher intake of milk also consumed higher amounts of fruit and vegetables and lower intakes of grain and meat (rich in carbohydrate and fat) as compared with those of non or rarely intake of milk. However, the observed inverse association of milk consumption on MetS persisted even after adjusting for the intake of different food groups.

In our study, subjects with higher milk consumption (≥1 serving/day in men and ≥2 servings/day in women) have more energy intake and a lower prevalence of MetS. Based on the assumption that individuals tend to misreport intakes of most reported foods and beverages to a similar degree and in the same direction [51], it is possible that higher absolute energy intakes usually tend to result in higher intakes of food group. Thus, the positive association between milk consumption and absolute energy intake may be observed in our study. Variation between individuals in absolute total energy intake is caused by body size, metabolic efficiency, physical activity, weight change, and etc. Thus, if energy intake is associated with disease, but not a direct cause, total energy intake may be confounder. It is generally accepted that energy adjustment is useful in mitigating the effects of measurement error in data collected using self-reported dietary assessment instruments, especially FFQ [51]. Therefore, to identify the association between milk consumption and the prevalence of MetS independent of energy intake, we adjusted for total energy intake in multivariable-adjusted model. 

Moreover, there was a gender difference of MetS prevalence under the similar energy intake and the association between milk consumption and the odd of MetS was stronger among women than among men. It is possible that the degree of completion of the FFQ was different between men and women. Women may be more precise and reliable than men in completing the FFQ, as traditionally in Korea, women generally prepare the meals and more knowledgeable about diet and food. Thus, the energy intake among men may be under-reporting in our study. Additionally, this may be in part attributable to residual confounding effects, particularly from smoking or alcohol drinking. Since the proportion of current smokers and drinkers was much higher in men than in women in our study, these lifestyle factors may attenuate the association between milk consumption and MetS among men. Another explanation may involve genetic differences between men and women in regards to diet-related pathology of MetS; specifically, the differences in sex chromosomes, sex-specific gene expression of autosomes, sex hormones, and their effect on organ systems may have played a role [52]. 

With regard to yogurt consumption, we did not observe any association with MetS among the HEXA population (OR: 1.03, 95% CI: 0.92–1.15, *p* for trend = 0.1708 in men, and OR: 0.98, 95% CI: 0.91–1.06, *p* for trend = 0.7160 in women). This may be due to the fact that the type of yogurt consumed in Korea is different from the conventional yogurt (i.e., Greek, not-curd) consumed in Western countries. The most popular yogurt in Korea is mostly water-based containing 13.8 g of sugar per 100 mL, higher than that of coke (11.0 g per 100 mL). Furthermore, it is given that dairy processing generates a variety of biochemical changes to milk consumption, including the loss of more labile constituents (e.g., vitamin C and enzymes), the removal of bioactive components (e.g., whey removal), and/or the addition of ingredients (e.g., sugar, artificial sweeteners, and flavorings) [53]. These may partly explain no association between yogurt consumption and the prevalence of MetS among our subjects.

Several limitations should be considered when examining the results of the current study. First, results from the current study cannot provide insight on the causation between milk consumption and MetS due to the cross-sectional design. However, the observed association did not change after we excluded subjects who reported histories of type 2 diabetes, hypertension, and dyslipidemia, which may have an effect on dietary change. Nonetheless, prospective studies on milk consumption and subsequent risk of MetS are warranted to support the findings of the current study. Second, the information on consumption of milk and dairy products was obtained from self-reported FFQ; thus, measurement errors in dietary assessment are inevitable and energy intake from FFQ may be less accurate when compared with that from dietary record [54]. Although we adjusted energy intake to mitigate the effects of measurement error in data collected using the self-reported dietary assessment method, there was still substantial biases and inaccuracies in self-reported energy intake. Therefore, it needs further studies about the association dietary factors and disease outcomes using objective and accurate measurement tools in the future [55]. Additionally, we did not obtain the information regarding milk subtypes (whole, low-fat, and skim) from our FFQ, on the basis that most Korean adults consume whole milk, which is most widely available and most popular in Korea. Therefore, we could not assess the effect of milk subtypes (whole, low-, and skimmed) with different fat content on the risk of MetS. Third, health-related behaviors (i.e., smoking, drinking, and physical activities) were ascertained based on self-reporting methods; thus, the present study may not be free of information bias. Finally, as in any observational study, although we carefully adjusted for the relevant confounders, we cannot entirely rule out the possibility of some unmeasured or residual confounding factors (e.g., dietary behavior and measurement error) associated with milk consumption as well as MetS. 

Despite these limitations, several strengths are worth mentioning in our study. Our study includes a considerably large number of Korean adults, and by using the HEXA-G database; it is a sample that has become more homogenous with increased internal validity. We have also used a validated FFQ and standardized procedures to collect data, and adjusted for potentially crucial confounders to parse out the independent association of milk consumption with MetS.

## 5. Conclusions

In conclusion, a higher consumption of milk was associated with the lower prevalence of overall MetS, elevated WC, hypertriglyceridemia, and lower HDL-C in Korean adults. On this basis, it is necessary to invest in strategies for improving eating habits, with a greater consumption of healthy foods including milk among measures taken for the prevention of MetS and of chronic non-communicable diseases. Additionally, we suggest that further research is needed among the general population and to confirm the results in prospective studies including clinical trials with a more precise diet-assessment method.

## Figures and Tables

**Table 1:** Baseline characteristics * by milk consumption, the Health Examinee Study-Gem (HEXA-G) study, 2004–2013.

	Milk Consumption					*p*-Value ^†^
	Non or Rarely	≤2/Week	3–6/Week	≥1/Day		
**Men (*N* = 43,786)**	18,972	11,588	5936	7186		
Age (years)	54.2 ± 8.2	52.7 ± 8.4	53.0 ± 8.4	53.9 ± 8.6		<0.0001
BMI (kg/m^2^)	24.4 ± 2.8	24.5 ± 2.7	24.4 ± 2.7	24.3 ± 2.7		0.0006
≥College or above, *n* (%)	6352 (34.0)	4495 (39.2)	2376 (40.6)	2764 (38.9)		<0.0001
Current smokers, *n* (%)	6257 (33.1)	3660 (31.7)	1736 (29.4)	2055 (28.7)		<0.0001
Current drinkers, *n* (%)	13,679 (72.2)	8606 (74.4)	4371 (73.8)	5101 (71.2)		0.5511
Regular exercisers, *n* (%)	10,152 (53.6)	6698 (57.9)	3584 (60.6)	4477 (62.4)		<0.0001
Dietary Intake						
Energy (kcal)	1730.8 ± 437.3	1835.1 ± 449.6	1960.5 ± 468.3	2054.2 ± 492.8		<0.0001
Carbohydrate (g)	312.8 ± 75.0	324.4 ± 76.5	338.3 ± 78.8	348.1 ± 83.3		<0.0001
Protein (g)	56.6 ± 20.5	61.5 ± 20.6	68.3 ± 21.8	73.8 ± 23.1		<0.0001
Fat (g)	25.5 ± 14.3	29.8 ± 14.5	34.9 ± 15.4	39.0 ± 16.0		<0.0001
Calcium (mg)	325.2 ± 169.7	380.7 ± 167.6	509.3 ± 183.1	666.0 ± 234.9		<0.0001
Grain (g)	643.9 ± 207.0	611.8 ± 192.3	572.9 ± 188.3	545.2 ± 189.1		<0.0001
Vegetable (g)	129.3 ± 102.0	129.3 ± 92.6	137.9 ± 95.3	138.3 ± 97.1		<0.0001
Fruit (g)	141.5 ± 144.0	140.6 ± 126.8	151.8 ± 132.0	160.7 ± 137.6		<0.0001
Meat (g)	41.0 ± 34.9	43.5 ± 31.6	42.1 ± 31.1	38.2 ± 30.7		<0.0001
						
	**Non or Rarely**	**≤2/Week**	**3–6/Week**	**1/Day**	**≥2/Day**	
**Women (*N* = 86,738)**	30,025	21,970	14,529	17,608	2606	
Age, years	53.1 ± 7.9	51.3 ± 7.7	51.5 ± 7.6	52.9 ± 7.7	52.2 ± 7.5	<0.0001
BMI, kg/m^2^	23.7 ± 3.0	23.6 ± 3.0	23.5 ± 2.8	23.6 ± 2.9	23.5 ± 2.8	<0.0001
≥ College or above, *n* (%)	4799 (16.2)	4578 (21.1)	3239 (22.6)	3639 (20.9)	585 (22.8)	<0.0001
Current smokers, *n* (%)	744 (2.5)	424 (1.9)	272 (1.9)	369 (2.1)	62 (2.4)	0.0291
Current drinkers, *n* (%)	8376 (28.0)	7144 (32.6)	4762 (33.0)	5368 (30.6)	787 (30.4)	<0.0001
Regular exercisers, *n* (%)	13,814 (46.1)	11,018 (50.2)	7949 (54.8)	9929 (56.5)	1565 (60.3)	<0.0001
Dietary Intake						
Energy (kcal)	1556.4 ± 456.2	1635.9 ± 466.3	1758.4 ± 484.5	1838.2 ± 489.5	2093.8 ± 555.7	<0.0001
Carbohydrate (g)	287.8 ± 81.9	295.9 ± 83.4	309.4 ± 85.4	318.1 ± 85.9	342.0 ± 95.7	<0.0001
Protein (g)	50.5 ± 20.0	54.2 ± 20.0	61.0 ± 21.3	65.7 ± 21.7	80.6 ± 27.3	<0.0001
Fat (g)	20.7 ± 12.8	24.4 ± 13.3	29.3 ± 14.3	32.6 ± 14.3	44.8 ± 17.1	<0.0001
Calcium (mg)	329.0 ± 184.2	380.2 ± 182.7	508.0 ± 194.4	633.5 ± 206.4	1053.1 ± 383.2	<0.0001
Grain (g)	572.4 ± 216.6	536.9 ± 201.8	497.0 ± 189.9	470.6 ± 184.9	387.8 ± 181.0	<0.0001
Vegetable (g)	150.7 ± 117.7	151.3 ± 110.9	158.9 ± 109.0	160.8 ± 109.4	181.0 ± 139.6	<0.0001
Fruit (g)	197.9 ± 184.1	202.8 ± 180.9	210.3 ± 171.1	219.5 ± 169.9	223.7 ± 190.7	<0.0001
Meat (g)	30.2 ± 30.2	33.5 ± 29.3	33.0 ± 28.7	30.4 ± 27.2	26.0 ± 24.5	<0.0001

* Values are means ± SD or *n* (%); nutrient intake values were energy adjusted using the residual method. ^†^ *p* values for differences between quintiles were calculated by chi-square tests for categorical variables and general linear regression for continuous variables. BMI: Body Mass Index.

**Table 2:** Odd ratios (OR) * and 95% confidence intervals (CI) of metabolic syndrome and components according to milk consumption; Ref. (Reference).

Metabolic Syndrome and Components	Milk Consumption					*p*-Trend ^†^
	Non or Rarely	≤2/Week	3–6/Week	≥1/Day		
**Men (*N* = 43,682)**	18,972	11,588	5936	7186		
MetS ^‡^	5715 (30.1) ^§^	3345 (28.9)	1671 (28.2)	1970 (27.4)		
	Ref.	0.94 (0.89–1.00)	0.93 (0.86–1.00)	0.92 (0.86–0.99)		0.0160
WC ≥ 90 cm	5607 (29.6)	3453 (29.8)	1703 (28.7)	2005 (27.9)		
	Ref.	0.97 (0.91–1.04)	0.9 (0.82–0.98)	0.91 (0.84–0.99)		0.0098
Serum TG ≥ 150 mg/dL	7765 (40.9)	4555 (39.3)	2241 (37.8)	2586 (36.0)		
	Ref.	0.91 (0.87–0.96)	0.87 (0.82–0.93)	0.84 (0.79–0.89)		<0.0001
Serum HDL-C ≤ 40 mg/dL	4622 (24.4)	2676 (23.1)	1279 (21.5)	1492 (20.8)		
	Ref.	0.95 (0.90–1.01)	0.88 (0.82–0.94)	0.83 (0.78–0.89)		<0.0001
BP ≥ 130/85 mmHg	10,192 (53.7)	5959 (51.4)	3060 (51.5)	3776 (52.5)		
	Ref.	0.94 (0.89–0.98)	0.94 (0.89–1.00)	0.99 (0.93–1.05)		0.7007
FB ≥ 100 mg/dL	6604 (34.8)	3946 (34.1)	2040 (34.4)	2559 (35.6)		
	Ref.	0.98 (0.93–1.04)	1.05 (0.97–1.14)	1.07 (0.99–1.16)		0.0500
						
	**Non or Rarely**	**≤2/Week**	**3–6/Week**	**1/Day**	**≥2/Day**	
**Women (*N* = 86,738)**	30,025	21,970	14,529	17,608	2606	
MetS	7053 (23.5)	6368 (29.0)	3203 (22.0)	4212 (23.9)	502 (19.3)	
	Ref.	0.88 (0.84–0.92)	0.86 (0.82–0.91)	0.85 (0.81–0.90)	0.68 (0.60–0.76)	<0.0001
WC ≥ 80 cm	13,168 (43.9)	8964 (40.8)	5635 (38.8)	7259 (41.2)	979 (37.6)	
	Ref.	0.97 (0.92–1.02)	0.91 (0.86–0.96)	0.94 (0.89–0.99)	0.79 (0.71–0.89)	0.0126
Serum TG ≥ 150 mg/dL	7579 (25.2)	4858 (22.1)	3051 (21.0)	3899 (22.1)	508 (19.5)	
	Ref.	0.93 (0.89–0.97)	0.87 (0.83–0.92)	0.86 (0.82–0.90)	0.76 (0.69–0.85)	<0.0001
Serum HDL-C ≤ 50 mg/dL	11,976 (39.9)	7667 (34.9)	4765 (32.8)	5884 (33.4)	724 (27.8)	
	Ref.	0.87 (0.84–0.91)	0.81 (0.77–0.84)	0.78 (0.75–0.81)	0.61 (0.56–0.67)	<0.0001
BP ≥ 130/85 mmHg	11,912 (39.7)	7702 (35.1)	5104 (35.1)	6677 (37.9)	893 (34.3)	
	Ref.	0.95 (0.90–0.99)	0.96 (0.91–1.02)	1.01 (0.96–1.07)	0.96 (0.86–1.08)	0.3906
FB ≥ 100 mg/dL	6139 (20.4)	4035 (18.4)	2689 (18.5)	3614 (20.5)	495 (19.0)	
	Ref.	0.97 (0.93–1.01)	1.01 (0.96–1.06)	1.07 (1.02–1.12)	1.04 (0.93–1.15)	0.0021

* Adjusted for age (40–49, 50–59, and 60–69), BMI (continuous; not adjusted for WC OR), recruitment site, education (≤elementary school, middle school, high school, ≥college, and unknown), smoking (never, past, current, and unknown), alcohol drinking (non, current, and unknown), regular exercisers (yes, no, and unknown), and total energy intake (quartiles). ^†^ Linear trends across categories of milk consumption were tested using the median consumption value for each category as a continuous variable. ^‡^ MetS: the presence of three or more of the following components: (1) waist circumference (WC) ≥90 cm in men and ≥80 cm in women; (2) high triglyceride (TG) level ≥ 150 mg/dL; (3) low high density lipoprotein cholesterol (HDL-C) level <40 mg/dL in men and <50 mg/dL in women or taking anticholesterol medication; (4) high blood pressure (BP) ≥ 130/85 mmHg or taking antihypertensive medicine; and (5) high fasting glucose (FB) level ≥ 100 mg/dL or taking medication to treat diabetes mellitus. ^§^ The number of cases (percentage).

## Supplementary Materials

The following are available online at www.mdpi.com/2072-6643/9/10/1102/s1, Table S1: STROBE Statement—Checklist of items that should be included in reports of cross-sectional studies.
