# Supplementary material for: Association between Milk Consumption and Metabolic Syndrome among Korean Adults: Results from the Health Examinees Study
Source: Nutrients. 2017 Oct 8;9(10):1102. doi: 10.3390/nu9101102 (PMC5691718; doi:10.3390/nu9101102)
Supplement: Supplementary file 1 [file nutrients-09-01102-s001.doc]

STROBE Statement—Checklist of items that should be included in reports of ***cross-sectional studies***

|  | Item No | Recommendation |
| --- | --- | --- |
| **Title and abstract** | 1 | (*a*) Indicate the study’s design with a commonly used term in the title or the abstract Page1, line 21-23 |
|  |  | (*b*) Provide in the abstract an informative and balanced summary of what was done and what was found Page1, line 28-37 |
| Introduction | | |
| Background/rationale | 2 | Explain the scientific background and rationale for the investigation being reported  Page1-2, line 41-65 |
| Objectives | 3 | State specific objectives, including any prespecified hypotheses Page2, line 66-78 |
| Methods | | |
| Study design | 4 | Present key elements of study design early in the paper Page2, line 81-84 |
| Setting | 5 | Describe the setting, locations, and relevant dates, including periods of recruitment, exposure, follow-up, and data collection Page2, line 81-91 |
| Participants | 6 | (*a*) Give the eligibility criteria, and the sources and methods of selection of participants Page2, line85-95 and figure1 |
| Variables | 7 | Clearly define all outcomes, exposures, predictors, potential confounders, and effect modifiers. Give diagnostic criteria, if applicable Page3-4, line 103-173 |
| Data sources/ measurement | 8* | For each variable of interest, give sources of data and details of methods of assessment (measurement). Describe comparability of assessment methods if there is more than one group Page3-4, line 103-154 |
| Bias | 9 | Describe any efforts to address potential sources of bias Page4, line 138-154 and page 4, line 160-172 |
| Study size | 10 | Explain how the study size was arrived at Page2, line81-95 |
| Quantitative variables | 11 | Explain how quantitative variables were handled in the analyses. If applicable, describe which groupings were chosen and why Page4, line 138-154 and page4, line 165-168 |
| Statistical methods | 12 | (*a*) Describe all statistical methods, including those used to control for confounding Page4, line 160-173 |
|  |  | (*b*) Describe any methods used to examine subgroups and interactions Not applicable |
|  |  | (*c*) Explain how missing data were addressed Page4, line 165-168 |
|  |  | (*d*) If applicable, describe analytical methods taking account of sampling strategy Not applicable |
|  |  | (*e*) Describe any sensitivity analyses Page4, line 172-173 |
| Results | | |
| Participants | 13* | (a) Report numbers of individuals at each stage of study—eg numbers potentially eligible, examined for eligibility, confirmed eligible, included in the study, completing follow-up, and analysed Page2, line 81-95 |
|  |  | (b) Give reasons for non-participation at each stage Page2, line 81-95 |
|  |  | (c) Consider use of a flow diagram Figure1 |
| Descriptive data | 14* | (a) Give characteristics of study participants (eg demographic, clinical, social) and information on exposures and potential confounders Table2 |
|  |  | (b) Indicate number of participants with missing data for each variable of interest |
| Outcome data | 15* | Report numbers of outcome events or summary measures Table3 and Page6, line 201 |
| Main results | 16 | (*a*) Give unadjusted estimates and, if applicable, confounder-adjusted estimates and their precision (eg, 95% confidence interval). Make clear which confounders were adjusted for and why they were included Table3 and Page4, line 157-172 |
|  |  | (*b*) Report category boundaries when continuous variables were categorized Not applicable |
|  |  | (*c*) If relevant, consider translating estimates of relative risk into absolute risk for a meaningful time period Not applicable |
| Other analyses | 17 | Report other analyses done—eg analyses of subgroups and interactions, and sensitivity analyses Page6, line 214-219 |
| Discussion | | |
| Key results | 18 | Summarise key results with reference to study objectives Page7, line 235-250 |
| Limitations | 19 | Discuss limitations of the study, taking into account sources of potential bias or imprecision. Discuss both direction and magnitude of any potential bias Page9, line 330-351 |
| Interpretation | 20 | Give a cautious overall interpretation of results considering objectives, limitations, multiplicity of analyses, results from similar studies, and other relevant evidence Page9, line 358-364 |
| Generalisability | 21 | Discuss the generalisability (external validity) of the study results Page9, line 352-364 |
| Other information | | |
| Funding | 22 | Give the source of funding and the role of the funders for the present study and, if applicable, for the original study on which the present article is based Not applicable |

*Give information separately for exposed and unexposed groups.

**Note:** An Explanation and Elaboration article discusses each checklist item and gives methodological background and published examples of transparent reporting. The STROBE checklist is best used in conjunction with this article (freely available on the Web sites of PLoS Medicine at http://www.plosmedicine.org/, Annals of Internal Medicine at http://www.annals.org/, and Epidemiology at http://www.epidem.com/). Information on the STROBE Initiative is available at www.strobe-statement.org.
